# Anti-Inflammatory Response in TNFα/IFNγ-Induced HaCaT Keratinocytes and Probiotic Properties of *Lacticaseibacillus rhamnosus* MG4644, *Lacticaseibacillus paracasei* MG4693, and *Lactococcus lactis* MG5474

**DOI:** 10.4014/jmb.2301.01028

**Published:** 2023-05-05

**Authors:** Ji Yeon Lee, Jeong, Yong Park, Yulah Jeong, Chang, Ho Kang

**Affiliations:** Mediogen Co., Ltd., Jecheon 27159, Republic of Korea

**Keywords:** Anti‐inflammation, cell‐free supernatant, lactic acid bacteria, probiotics, keratinocytes

## Abstract

Atopic dermatitis (AD) is a chronic inflammatory disease caused by immune dysregulation. Meanwhile, the supernatant of lactic acid bacteria (SL) was recently reported to have anti-inflammatory effects. In addition, HaCaT keratinocytes stimulated by tumor necrosis factor alpha (TNF-α) and interferon gamma (IFN-γ) are widely used for studying AD‐like responses. In this study, we evaluated the anti-inflammatory effects of SL from lactic acid bacteria (LAB) on TNF-α/IFN-γ-induced HaCaT keratinocytes, and then we investigated the strains’ probiotic properties. SL was noncytotoxic and regulated chemokines (macrophage-derived chemokine (MDC) and thymus and activation-regulated chemokine (TARC)) and cytokines (interleukin (IL)‐4, IL‐5, IL‐25, and IL‐33) in TNF-α/IFN-γ-induced HaCaT keratinocytes. SL from *Lacticaseibacillus rhamnosus* MG4644, *Lacticaseibacillus paracasei* MG4693, and *Lactococcus lactis* MG5474 decreased the phosphorylation of nuclear factor-κB (NF-κB) and mitogen-activated protein kinase (MAPK). Furthermore, the safety of the three strains was demonstrated via hemolysis, bile salt hydrolase (BSH) activity, and toxicity tests, and the stability was confirmed under simulated gastrointestinal conditions. Therefore, *L. rhamnosus* MG4644, *L. paracasei* MG4693, and *Lc. lactis* MG5474 have potential applications in functional food as they are stable and safe for intestinal epithelial cells and could improve atopic inflammation.

## Introduction

Atopic dermatitis (AD) is a common and chronic, relapsing inflammatory skin disease with itchy, pruritic lesions [1]. In particular, since the symptoms of AD appear in visible places such as the hands and ears, they reduce the patient's quality of life and seriously affect their mental health [2]. Over the past decade, AD has markedly increased in prevalence in children and adults, and the resulting economic burden of managing this disease is reported to be significant [3, 4]. Synthetic drugs approved by the FDA to treat AD have been developed, but they cause side effects, such as conjunctivitis and headache [5]. Therefore, for the treatment of AD, preventive agents using natural materials that are safe and free of side effects are urgently needed.

The epidermis of the skin, which is mainly composed of keratinocytes, is the outermost part of the human body and acts as a physical barrier against various triggers [6]. Keratinocytes primarily modulate the immune system in the skin [7]. Stimuli that cause inflammation in keratinocytes include ultraviolet B (UVB), interleukin (IL)‐1α, lipopolysaccharide (LPS), tumor necrosis factor alpha (TNF-α), and interferon gamma (IFN-γ) [8]. Keratinocytes produce chemokines (macrophage-derived chemokine (MDC), thymus- and activation-regulated chemokine (TARC)) and inflammatory cytokines (thymic stromal lymphopoietin (TSLP), IL-4, IL-25, and IL-33), which contribute to an inflammatory response in the skin [9]. When hyper‐immunity occurs in keratinocytes due to harmful triggers, chronic inflammation is induced, which causes atopic dermatitis (AD) accompanied by severe itching. Thus, an immune imbalance in keratinocytes has been implicated in AD pathogenesis [10]. A number of natural products have been reported for their anti-inflammatory efficacy related to atopic‐like responses; however, studies on lactic acid bacteria (LAB) in keratinocytes are still lacking [11].

LAB are a group of gram-positive bacteria, including *Lactobacillus* and *Bifidobacterium*, that are used as probiotics [12]. Probiotics are generally defined as safe microorganisms that have health‐promoting effects on the host when ingested in adequate amounts [13]. LAB have recently been reported to be effective not only in restoring gut microbiome balance, but also in improving skin health [14]. LAB are known to play a role in reducing AD severity by regulating immune status through barrier improvement [15]. Considerable evidence has revealed that metabolites, cell wall fragments, and dead cells of LAB improve skin health [16]. Among the metabolites present in the supernatant of LAB (SL), short-chain fatty acids (SCFAs; mainly acetate, propionate, and butyrate) are beneficial for skin diseases such as AD, ichthyosis, acne, and psoriasis [15, 16].

Therefore, in this study we investigated the effect of SL on the production of chemokine, including TARC and MDC, and the mRNA expression of IL4, IL‐5, IL‐25, and IL‐33 in TNF-α/IFN-γ-induced HaCaT keratinocytes. We also evaluated the effect of SL on the NF-κB and MAPK signaling pathways in these keratinocytes. Finally, we analyzed the SCFAs and examined the probiotic properties, stability, and safety of the strains with anti-inflammatory activity.

## Materials and Methods

### Preparation of LAB Strains

All of the LAB strains used in this study were provided by Mediogen Co., Ltd. (Korea) and identified using 16S rRNA gene sequencing (SolGent Co., Ltd., Korea). Isolates were originated from human and/or fermented foods, and the details with NCBI accession numbers are listed in Table 1. LAB were cultured in De Man, Rogosa, and Sharpe (MRS) broth (BD Biosciences, USA) in an anaerobic chamber at 37°C. After 18 h, the SL was collected via centrifugation (4,000 ×*g*) for 15 min at 4°C, and then filtered using a 0.2‐μm polytetrafluoroethylene (PTFE) membrane (ADVANTEC, Japan) [17].

### Cell Culture

HaCaT keratinocytes (provided from the School of Cosmetic Science and Beauty Biotechnology, Semyung University, Korea) and HT‐29 cells (Korea Cell Line Bank, Korea) were cultured in Dulbecco’s modified Eagle’s medium (DMEM; Gibco, USA) containing 10% heat-inactivated fetal bovine serum (FBS; Gibco) and 1%penicillin and streptomycin (P/S; Gibco) [6]. The cells were maintained at 37°C under a 5% CO_2_ incubator and split every 2–3 days.

### Cell Viability

Cell viability was determined using the 3‐(4,5‐dimethyl‐2‐thiazolyl)‐2,5‐diphenyletrazolium bromide (MTT) assay [18]. HaCaT keratinocytes were seeded at a density of 1.5 × 10^4^ cells/well in 96‐well plates. SL from LAB was pre-treated for 1 h and then co-treated with or without TNF-α/IFN-γ (10 ng/ml, each) for 24 h. After removing the culture media, 0.25 mg/ml of MTT solution (Sigma-Aldrich, USA) was added to each well for 2 h. The formazan crystals formed by the MTT solution were dissolved in DMSO (150 μl). Absorbance at 550 nm was measured using a microplate spectrophotometer (Biotek, USA).

### Enzyme‐Linked Immunosorbent Assays (ELISA)

The levels of CCL17/TARC and CCL22/MDC were quantified using the Human CCL17/TARC Human and CCL22/MDC DuoSet ELISA Kit (R&D Systems, USA) according to the manufacturer’s instructions. The optical density at 450 nm was measured using a microplate spectrophotometer (Biotek).

### Preparation of mRNA and Real‐Time Polymerase Chain Reaction (PCR)

The mRNA was isolated using NucleoZol (MACHEREY‐NAGEL, Gutenberg, Hoerdt Cedex, France) following the manufacturer’s protocol. The cDNA was prepared from the isolated mRNA using the Maxime RT PreMix (iNtRON, Korea). The mRNA expression was analyzed using the CFX96 System (Bio‐Rad, USA) with the AmfiSure qGreen Q‐PCR Master Mix (Gendepot, USA) and the following primers: IL‐4 (forward; f): 5'‐AACAGCCTCACAGAGCAGAAGAC‐3', IL‐4 (reverse; r): 5'‐GTGTTCTTGGAGGCAGCAAAG‐3'; IL‐5 (f): 5'‐TCTACTCATCGAACTCTGCTGA‐3', IL‐5 (r): 5'‐CCCTTGCACAGTTTGACTCTC‐3'; IL‐25 (f): 5'‐TTGTTTGTTTACTCATCACTCAG‐3', IL‐25 (r): 5'‐TCCTCCTCAGAATCATCCA‐3'; and IL‐33 (f): 5'‐TGTCACATTGGGCAAAGTT‐3', IL‐33 (r): 5'‐CAGTAAGCAGTGTTATCAGGAA‐3'. The mRNA expression was normalized using GAPDH (f): 5'‐ACCCACTCCTCCACCTTTG‐3' and GAPDH (r): 5'-ACCCACTCCTCC ACCTTTG‐3'. Relative quantitative expression was analyzed using the 2^‐ΔΔCT^ method and determined as a fold change of the TNF-α/IFN-γ-treated control [19].

### Protein Extraction and Western Blotting

Protein lysates were obtained using radioimmunoprecipitation assay (RIPA) cell lysis buffer (Gendepot) containing phosphatase and protease inhibitors (Gendepot). The extracted proteins were quantified at 1 μg/μl using Bradford (Coomassie) reagent (Gendepot). The protein samples were prepared in a 5X sample buffer (iNtron) and heated at 85°C for 10 min. Western blotting was performed using sodium dodecyl sulfate-polyacrylamide gel electrophoresis (SDS‐PAGE) [20]. The samples were loaded onto 10% Tris–glycine gels and electrophoresed in Tris/Glycine/SDS buffer (Bio-Rad). The separated proteins were transferred to polyvinylidene difluoride (PVDF) membranes (Millipore, USA) and washed with TBS-Tween buffer (TBS-T, Gendepot). After blocking with the Smart‐Block 5 min‐Fast Blocking Buffer (Biomax, Republic of Korea), the membranes were incubated with primary antibodies, phosphorylated (p)‐IκB, IκB, p‐nuclear factor kappa B (NF‐κB) p65, extracellular signal‐regulated kinases (ERK), p‐ERK, p‐c‐Jun N‐terminal kinase (JNK), p38, and p‐p38 (Cell Signaling Technology, USA), and NF-κB p65, JNK, and β‐actin (USA), diluted to 1:1000 overnight at 4°C. After washing with TBS‐T, membranes were incubated with HRP‐conjugated secondary antibodies (1:5000, Gendepot) for 1 h. The western blot images were developed using the LuminoGraph III Lite (Atto Corp., Japan) with the EzWest Lumi Plus (Atto), and density graph analysis was performed using the CS Analyzer 4 (Atto).

### Gas Chromatograpy/Mass Spectrometry (GC/MS) Analysis of SCFAs

SCFAs in SL were analyzed using GC–MS (QP2020 NXW/ORP230, Shimadzu, Japan) equipped with a Stabilwax-DA column (60 m × 0.32 mm × 0.25 μm, Shimadzu) according to a previous study [21]. GC analysis was conducted in splitless mode using helium (flow rate, 2 ml/min) as the carrier gas. The oven temperature was held at 50°C for 2 min and then ramped up to 200°C at 10°C/min. This temperature was held for 5 min. The ionization was operated using the following settings: electron energy = 70 eV, ion source heater temperature = 200°C and 150°C, and scan speed = 0.2 s/scan. The analytes were monitored in the selected ion monitor (SIM) mode. Acetic acid, propionic acid, and butyric acid (Sigma-Aldrich) were used as standards and the quantitative analysis in CFS was calculated using the standard curve.

### Profiling of Carbohydrate Fermentation and Enzymatic Activity

Carbohydrate fermentation of LAB was measured using the API 50 CHL Kit (BioMerieux, France) [22]. Briefly, LAB strains were grown on MRS agar (BD Biosciences) plates for 18 h, and the colonies were adjusted in API suspension medium (BioMerieux) to 2.0 McFarland. The suspended LAB strains were inoculated into strips and incubated at 37°C for 48 h. Meanwhile, the enzymatic activities of LAB were confirmed using the API ZYM Enzyme System (BioMerieux) [23]. After streaking on MRS medium, a colony was cultured at 37°C for 18 h, and the turbidity was adjusted to 5.0–6.0 McFarland in API suspension medium (BioMerieux). The suspension medium was dispensed on each enzyme of the API ZYM strip and incubated at 37°C for 4 h. The results were confirmed by the color changes.

### Cytotoxicity to HT‐29 Cells

Cytotoxicity was estimated using the MTT assay [17]. HT‐29 cells were seeded at a density of 2.5 × 10^4^ cells/well in 96‐well plates. LAB were treated at 10^6^, 10^7^, and 10^8^ CFU/ml for 24 h, and then the culture medium was removed and MTT solution (0.25 mg/ml) was added to each cell for 2 h. The formazan crystals, formed by MTT solution, were dissolved in DMSO (150 μl). Absorbance at 550 nm was measured using a microplate spectrophotometer (Biotek).

### Hemolytic and Bile Salt Hydrolase (BSH) Activity

Hemolytic activity was determined using Columbia agar (Oxoid, UK) containing 5% sheep blood (MBcell, Korea) [24]. LAB strains were streaked and cultured at 37°C for 48 h. Hemolysis was observed as halo zones around the colony: (1) alpha hemolysis appeared green in the medium, suggesting partial hemolysis; (2) beta hemolysis showed a clear zone, indicating destroyed red blood cells; and (3) gamma hemolysis did not show any reaction in the surrounding medium, indicating a lack of hemolysis [25]. Meanwhile, BSH enzyme activity of the LAB strains was determined according to a previous report [26]. Briefly, each strain was cultured on MRS agar containing 0.5% taurodeoxycholic acid hydrate (Sigma-Aldrich) and incubated at 37°C under anaerobic conditions for 48 h. BSH activity was assessed with the appearance of a precipitation zone surrounding the colony.

### Survival in Gastrointesinal Tract (GIT) Conditions

Survival under conditions similar to those in the GIT environment was assessed as previously described [27]. The viability of LAB was measured after 2 h in PBS (pH 2.5) with 0.3% pepsin (Sigma-Aldrich) as gastric fluids at 37°C, and after 2.5 h in PBS (pH 7.4) with 1% pancreatin‐bile salt (Sigma-Aldrich) as intestinal fluids at 37°C. The live colony count was performed using an MRS agar plate.

### Adhesion to HT‐29 Cells

The adhesion test to HT‐29 cells, a human colon adenocarcinoma cell line, was performed as reported previously with some modifications [28]. Briefly, HT‐29 cells were sub‐cultured at 1.5 × 10^5^ cells/well in 12‐well plates and grown at 37°C in a humidified atmosphere of 5% CO_2_ until they formed a monolayer. The cells were then treated with LAB (1 × 10^8^ CFU/ml) for 4 h. After treatment, the HT‐29 cells were washed three times with PBS and then lysed with 1 ml of PBS. The cell lysate was diluted with buffered peptone water (Oxoid), plated on MRS agar, and incubated at 37°C for 48 h. Adhesion ability (%) was calculated using the following equation: log (adherent counts of LAB) CFU/ml/log (initial counts of LAB) CFU/ml × 100.

### Statistical Analysis

Results are expressed as mean ± standard error of the mean (SEM). Statistical significance was analyzed using one-way analysis of variance (ANOVA) followed by post hoc verification with Duncan’s multiple comparison tests (SPSS Statistics ver. 21, IBM, USA).

## Results

### SL Suppressed Chemokines in TNF-α/ IFN-γ‐Induced HaCaT Keratinocytes

**Fig. 1:**
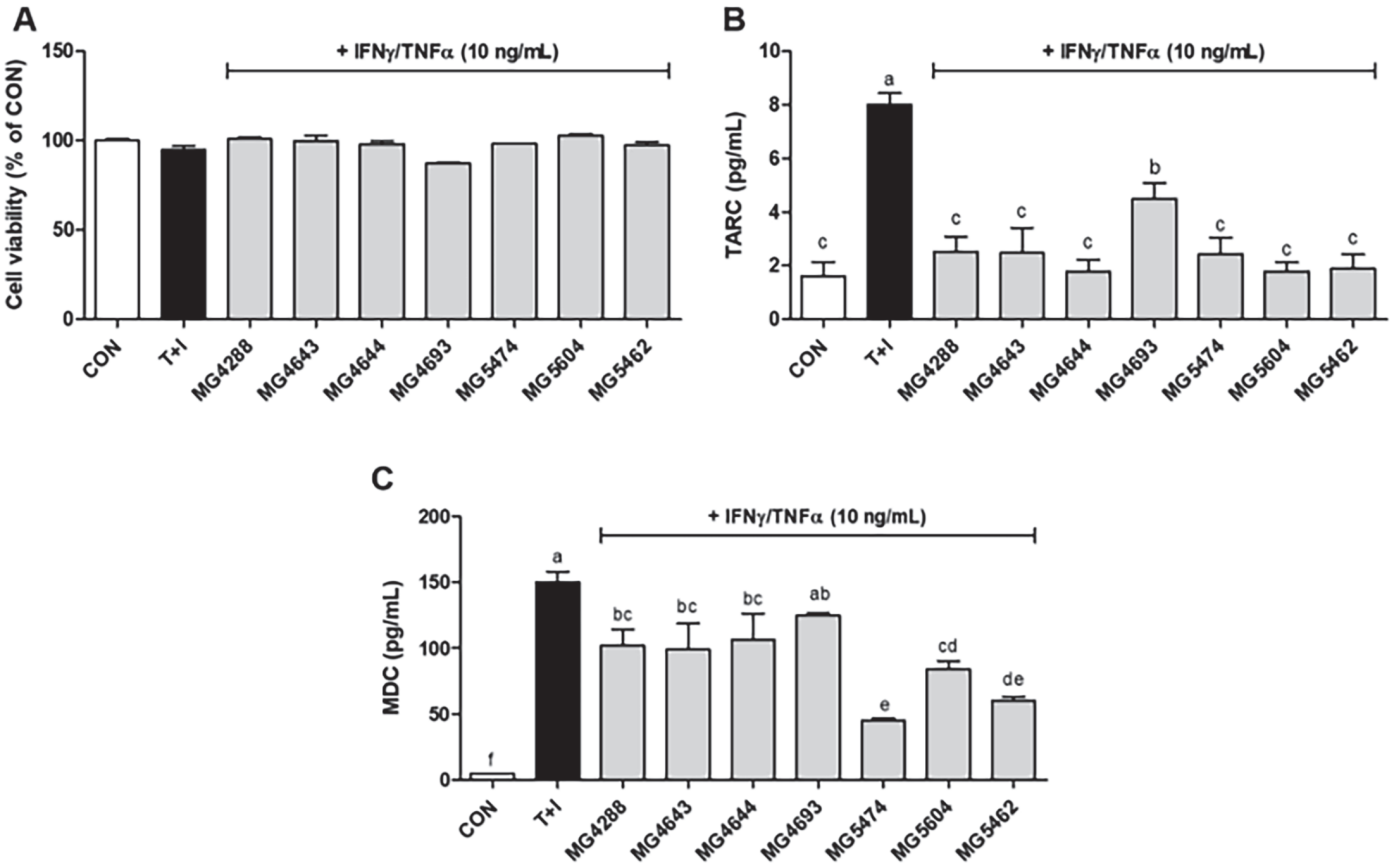
Effect of SL (5%) from LAB on cell viability (A), expression of TARC (B) and MDC (C) in TNF-α/IFN- γ‐induced HaCaT keratinocytes. The bars indicate the mean ± SEM. Different letters (a–f) show significant difference at *p* < 0.05 (*n* = 3). *T+I, TNF-α/IFN-γ only treated group.

HaCaT keratinocytes were treated with various concentrations of SL to optimize the concentration. The results showed no toxicity at 5% SL (Fig. 1A). As shown in Fig. 1B, 5% SL was not cytotoxic to TNF-α/IFN-γ‐induced HaCaT keratinocytes; therefore, further experiments were conducted at this concentration. TNF-α/IFN-γ-treated HaCaT keratinocytes showed significantly upregulated TARC levels (Fig. 1C). Conversely, treatment with SL from LAB and TNF-α/IFN-γ markedly decreased TARC levels (*p* < 0.05). Similarly, MDC levels were significantly upregulated (*p* < 0.05) by TNF-α/IFN-γ on HaCaT keratinocytes, but were reduced upon treatment with SL (*p* < 0.05), as shown in Fig. 1D. In particular, several strains showed reduced efficacy in TARC than in MDC, and *Lc. lactis* MG5474 strain showed the lowest production of TARC (0.26‐fold of TNF-α/IFN-γ) and MDC (0.30‐fold of TNF-α/IFN-γ).

### Inhibition of Cytokines Related to Immune Response by SL in TNF-α/IFN-γ‐Induced HaCaT Keratinocytes

**Fig. 2:**
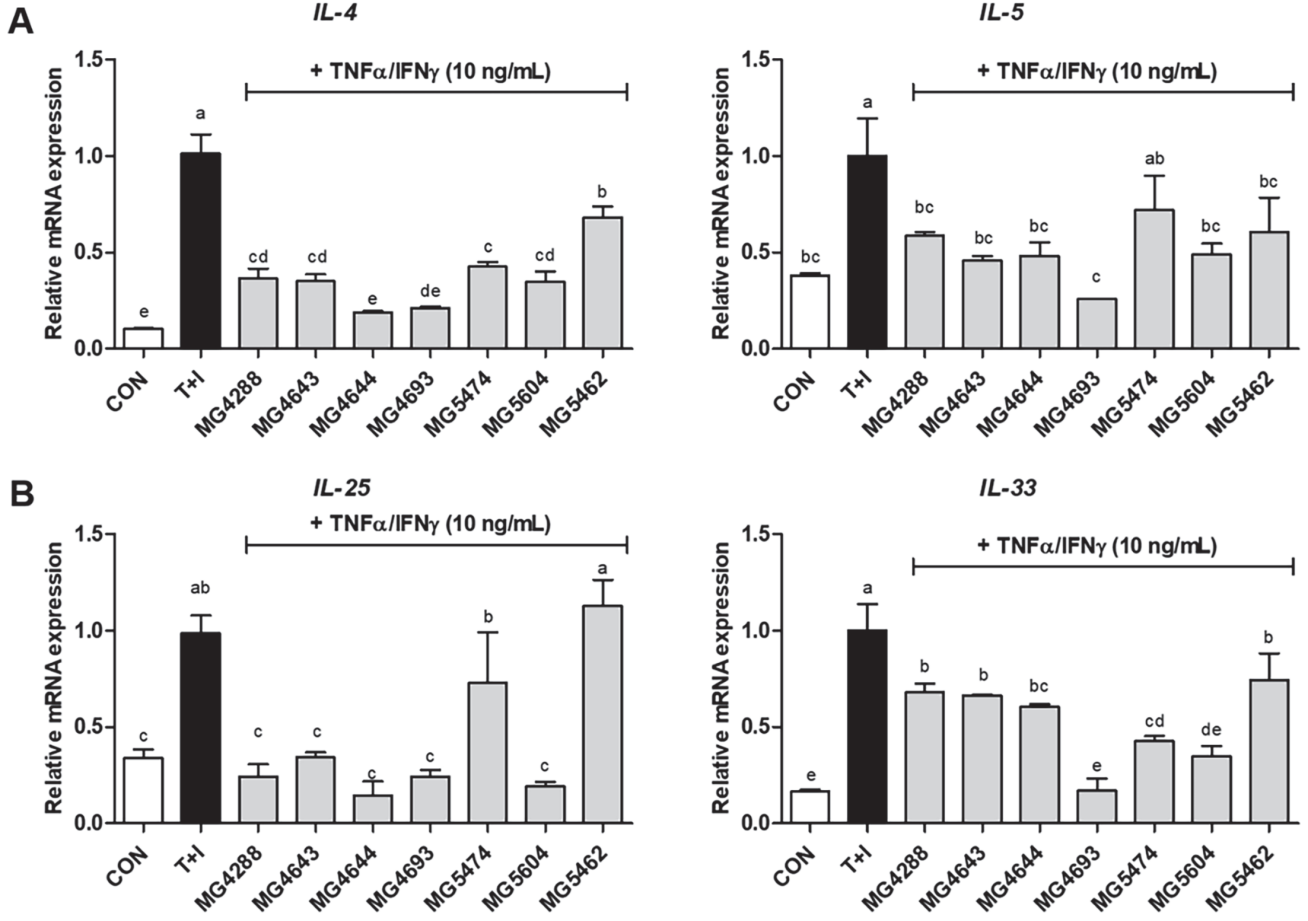
Effect of SL (5%) from LAB strains on *IL‐4*, *IL‐5* mRNA expression (A), TSLP (B) and IL‐25, IL‐33 mRNA expression (C) in TNF-α/IFN-γ‐induced HaCaT keratinocytes. The bars indicate the mean ± SEM. Different letters (a–e) show significant difference at *p* < 0.05 (*n* = 3). *T+I, TNF-α/IFN-γ only treated group.

To determine the effect of different SL on cytokines, IL‐4, IL‐5 , IL‐25, IL‐33, and thymic stromal lymphopoietin (TSLP) were analyzed using real-time PCR and ELISA. As shown in Fig. 2A, IL‐4 and IL‐5 mRNA levels were significantly elevated in TNF-α/IFNγ-induced HaCaT keratinocytes (*p* < 0.05); however, these elevations were reduced by most SL. In addition, TSLP levels were markedly increased in TNF-α/IFN-γ-induced HaCaT keratinocytes (Fig. 2B). Conversely, SL decreased the TSLP levels in TNF-α/IFN-γ-induced HaCaT keratinocytes (*p* < 0.05). Among the SL, the effect of the SL of *L. paracasei* MG4693 on IL‐4 and IL‐5 mRNA levels (0.21-and 0.26‐fold of TNF-α/IFN-γ, respectively) and TSLP levels (0.34‐fold of TNF-α/IFN-γ) was inhibited more profoundly than that of other SL. Subsequently, we measured the mRNA levels of IL‐25 and IL‐33 in TNF-α/IFN-γ‐induced HaCaT keratinocytes using RT‐qPCR (Fig. 2C). The IL‐25 and IL‐33 mRNA levels were increased in TNF-α/IFN-γ-induced HaCaT keratinocytes, while these mRNA levels were decreased in most SL-treated keratinocytes. Notably, the effect of the SL of *L. paracasei* MG4693 (0.24 and 0.17‐fold of TNF-α/IFN-γ) was much more prominent than that of other SL.

### Effect of SL on NF‐κB/MAPK Signaling Pathways in TNF-α/ IFN-γ‐Induced HaCaT Keratinocytes

**Fig. 3:**
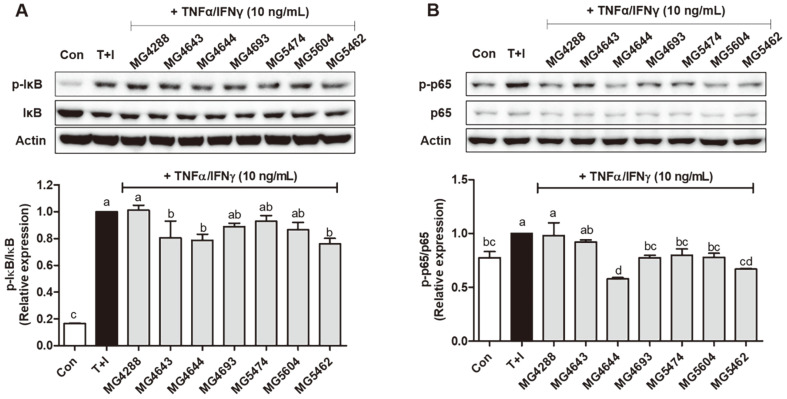
Effect of SL (5%) from LAB strains on the phosphorylation of nuclear factor kappa B (Nf‐κB) signaling pathway in TNF-α/IFN-γ‐induced HaCaT keratinocytes. The protein expression of IκB (**A**) and Nf‐κB (**B**) were normalized to non‐phosphorylated protein. The bars indicate the mean ± SEM. Different letters (a–d) show significant difference at *p* < 0.05 (*n* = 3). *T+I, TNF-α/IFN-γ only treated group.

**Fig. 4:**
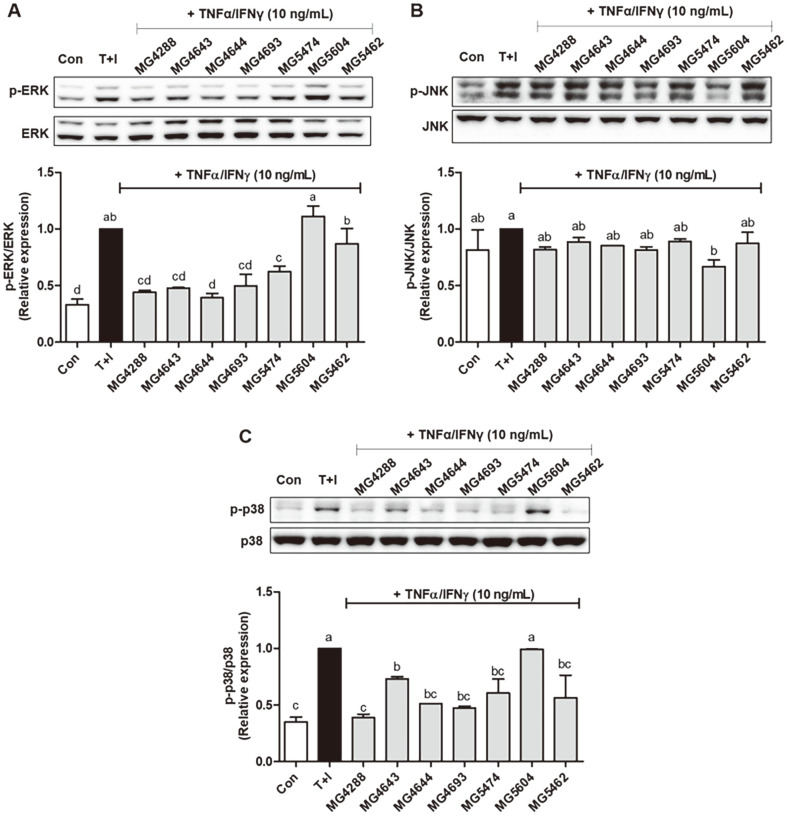
Modulation of the mitogen‐activated protein kinase (MAPK) signaling by SL (5%) from LAB strains in TNF-α/IFN-γ‐induced HaCaT keratinocytes. Representative western blot images of p‐ERK/ERK (**A**), p‐JNK/JNK (**B**), and p‐p38/p38 (**C**) expression was shown. The protein expression was normalized to non‐phosphorylated protein. The bars indicate the mean ± SEM. Different letters (a–d) show significant difference at *p* < 0.05 (*n* = 3). *T+I, TNF-α/IFN-γ only treated group.

To investigate the effect of SL on the NF-κB signaling pathway, p‐IκB and p‐NF‐κB were analyzed using western blotting (Fig. 3). Increased p‐IκB and p‐NF‐κB levels were observed in TNF-α/IFN-γ-induced HaCaT keratinocytes (*p* < 0.05). These levels were decreased after treatment with SL of *L. rhamnosus* MG4644 and *L. reuteri* MG5462 (*p* < 0.05). Investigation of MAPK activation in the presence of SL and TNF-α/IFNγ in HaCaT keratinocytes was also conducted, and as shown in Fig. 4, the phosphorylation of ERK and p38 stimulated by TNF-α/IFN-γ was signiﬁcantly reversed by most SL (Figs. 4A and 4C), whereas the phosphorylation of JNK was not signiﬁcantly different (Fig. 4B). These results suggest that SL, particularly from *L. rhamnosus* MG4644, can inactivate the NF‐κB/MAPK signaling pathways.

### Analysis of SCFAs in SL from *L. rhamnosus* MG4644, *L. paracasei* MG4693, and *Lc. lactis* MG5474

To analyze SCFAs, which are major metaboiltes of probiotics produced by each strain, the contents of acetic, propionic and butyric acid in SL were measured using GC-MS (Table 2). The calibration curve formulas of acetic, propionic and butyric acid were confirmed as y = 35590x + 44682, y = 105472x + 10648, and y = 241306x + 3248.1, respectively, and through this, quantitative analysis of SCFA in SL was performed. All three strains were confirmed to have high levels of acetic acid content; however, *L. rhamnosus* MG4644 showed the highest content of propionic (1.49 ± 0.06 mg/l) and butlyric acid (5.37 ± 0.04 mg/l) than other strains, while *L. paracasei* MG4693 showed the highest level of acetic acid (3112.43 ± 79.35 mg/l) and total SCFAs (3118.55 ± 79.68 mg/l).

### Probiotic Properties of *L. rhamnosus* MG4644, *L. paracasei* MG4693, and *Lc. lactis* MG5474

As shown in the carbohydrate fermentation profiles, all three strains fermented various sugars (Table 3). We found that all strains utilized L-arabinose, methyl-β-D-xyloside, D-galactose, D-glucose, D-fructose, methyl-α-D-glucoside, arbutin, esculin, salicin, D-cellobiose, D-maltose, D-melibiose, and D-sucrose, and did not utilize glycerol, D-xylose, L-xylose, D-adonitol, D-sorbitol, starch, glycogen, D-turanose, D-tagatose, L-fucose, D-arabitol, gluconate, and 2-keto-gluconate. Each strain differed in its ability to utilize the remaining carbohydrates. Based on these results, the three strains were identified as *L. rhamnoses* MG4644, *L. piracies* MG4693, and *Lc. lactis* MG5474 using the API 50 CHL profile along with 16S rRNA gene sequencing. All three strains were examined using an API ZYM kit and exhibited most of the enzymatic activities, except trypsin, β-glucuronidase, and α‐mannosidase, as listed in Table 4. Among the unused enzymes, β-glucuronidase has been reported to play an important role in cancer development by reactivating carcinogens [29]. Thus, none of the three strains showed β-glucuronidase activity and may not cause cancer.

### Safety of *L. rhamnosus* MG4644, *L. paracasei* MG4693, and *Lc. lactis* MG5474

**Fig. 5:**
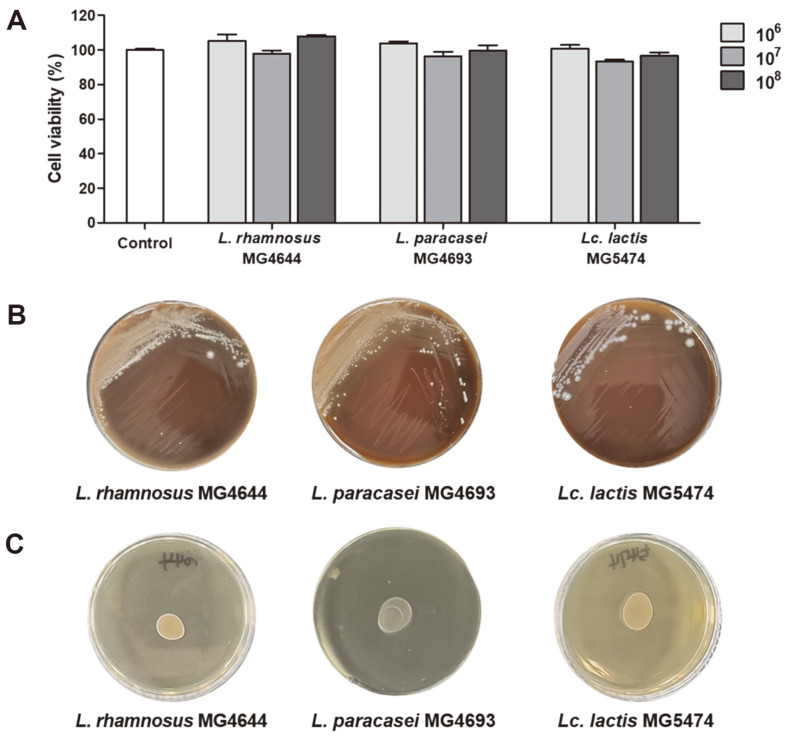
Hemolysis (A), BSH activity (B), and cytotoxicity of *L. rhamnosus* MG4644, *L. paracasei* MG4693, and *Lc. lactis* MG5474 in HT‐29 cells (C). HT‐29 cells were treated with *L. rhamnosus* MG4644, *L. paracasei* MG4693, and *Lc. lactis* MG5474 (10^6^, 10^7^, and 10^8^ cells/ml). Data are presented as the mean±SEM (*n*=3), and have no statistical significance.

To confirm the safety of *L. rhamnosus* MG4644, *L. paracasei* MG4693, and *Lc. lactis* MG5474, which have anti‐inflammatory effects in keratinocytes, cytotoxicity in treating HT‐29 cells, hemolysis, and BSH activity were evaluated. The three strains (10^6^–10^8^ CFU/ml) showed no cytotoxicity (≥ 90%) in HT‐29 cells (Fig. 5A). Moreover, the three strains exhibited gamma‐hemolysis, indicating no hemolytic effect on the host, and no BSH activity was observed (Figs. 5B and 5C).

### Stability in Simulated GIT and Adhesion to HT‐29 Cells of *L. rhamnosus* MG4644, *L. paracasei* MG4693, and *Lc. lactis* MG5474

**Fig. 6:**
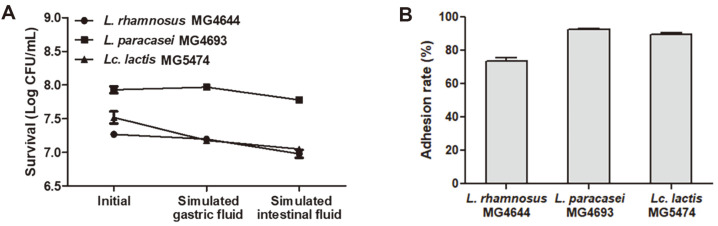
Cytotoxicity in HT‐29 cells (A), hemolysis (B), and BSH activity (C) of *L. rhamnosus* MG4644, *L. paracasei* MG4693, and Lc. lactis MG5474. HT‐29 cells were treated with *L. rhamnosus* MG4644, *L. paracasei* MG4693, and *Lc. lactis* MG5474 (10^6^, 10^7^, and 10^8^ cells/ml). Data are presented as the mean ± SEM (*n* = 3), and have no statistical significance.

The stability in simulated GIT of *L. rhamnosus* MG4644, *L. paracasei* MG4693, and *Lc. lactis* MG5474, which showed an anti‐inflammation effect, was determined (Fig. 6A). In the simulated gastric fluid, all strains showed a high survival rate of 95% or more. Furthermore, *L. rhamnosus* MG4644, *L. paracasei* MG4693, and *Lc. lactis* MG5474 showed viabilities of 96, 98.2, and 93.7% in simulated GIT condition. To determine the adhesion of the three strains to HT‐29 cells, the plate-counting method was performed. *L. rhamnosus* MG4644, *L. paracasei* MG4693, and *Lc. lactis* MG5474 were adherent to HT‐29 cells at 73.72, 92.49, and 90.34% compared to the initial colony number (Fig. 6B).

## Discussion

The gut-skin axis is currently being investigated based on studies showing that the immune regulation ability of the gut microbiome affects this distant organ [30]. Studies have reported that the skin and intestines have many similarities, and that skin conditions can be affected by changes in gut microbes [30]. The intestinal microflora acts on AD in two directions: (1) directly by producing metabolites such as SCFAs and delivering them to the skin through the blood vessels, and/or (2) indirectly by controlling the microbiota, which helps balance the immune system [31]. In a clinical study, *L. rhamnosus*, *L. reuteri*, *L. acidophilus*, *Bifidobacterium bifidum*, and *Bi. lactis* were reported to improve AD [32]. As an in vitro model, HaCaT keratinocytes are widely used because they are easy to use and are efficient in providing potentially meaningful results [33]. In particular, the synergistic action of TNF-α and IFN-γ is widely used as a skin inflammation model [34]. TNF-α/IFN-γ treatment in HaCaT keratinocytes is commonly employed to study AD‐like responses for evaluating the efficacy of functional foods or drugs [35]. In the current study, to discover LAB strains as functional probiotic candidates with potential efficacy to inhibit atopic‐like response, we evaluated the anti‐inflammatory effect of SL from LAB strains on chemokine and cytokine levels and NF‐κB/MAPK signaling pathways in keratinocytes, and assessed their stability and safety as probiotics.

The stimulation of TNF-α and IFN-γ results in the expression of many cytokines (IL family and TSLP) and chemokines (MDC and TARC) in keratinocytes [36]. TARC and MDC, which are specific ligands for the CC motif chemokine ligand 4 (CCR4) expressed by Th2 cells, have been reported to correlate with the pathogenesis of AD. In our study, most SL downregulated MDC and TARC levels, which were elevated by TNF-α/IFN-γ in keratinocytes. The incidence of AD-like inflammation can be improved by regulating cytokines involved in T-helper cell type (Th)-2 and Th-1/Th-2 ratio [15]. Inhibition of Th2 cytokines such as IL-4 and IL-5 is known to block the progression of AD-like inflammation [11]. TSLP, which induces Th2 differentiation and produces Th2 cytokines, contributes to pruritus in keratinocytes of patients with AD [10]. In the present study, the mRNA expression of IL-4, IL-5, and TSLP were most inhibited by *L. rhamnosus* MG4644 and *L. paracasei* MG4693. In addition, TSLP can control the generation of chemokines, such as MDC and TARC [37]. Our results suggest *Lc. lactis* MG5474, *Lc. lactis* MG5604, and *L. reuteri* MG5462 suppressed MDC and TARC by reducing TSLP expression. IL-25 and IL-33 prevent Th1 immune response and affect the secretion of Th2 cytokines, resulting in an imbalance in the Th1/2 ratio [10, 38]. In the present study, all SL of LAB, except for *L. reuteri* MG5462, effectively suppressed the mRNA expression of IL-25 and IL-33. Probiotics and/or compounds from various natural products prevent AD-like inflammation by modulating chemokines and cytokines related to Th-2 differentiation and Th-1/Th-2 ratio [11, 15]. Therefore, *L. rhamnosus* MG4288, *L. rhamnosus* MG4643, *L. rhamnosus* MG4644, *L. paracasei* MG4693, *L. lactis* MG5474, and *Lc. lactis* MG5604 may protect against AD-like inflammation.

Activation of NF-κB and MAPKs signaling pathways related to inflammation in keratinocytes is induced by cross-linking with TNF-α and IFN-γ [9]. NF-κB, stimulated by the degradation of IκB, is a major transcription factor involved in the inflammatory responses in AD [35]. Phosphorylated NF-κB translocates to the nucleus and produces cytokines and chemokines to regulate inflammatory and immune responses [36]. In our results, SL significantly diminished p‐IκB and p‐NF‐κB increased by TNF-α/IFN-γ in keratinocytes. Furthermore, NF-κB is regulated by the MAPK signaling pathway [36]. A previous study reported that inhibition of p‐p38 and p‐ERK attenuates skin inﬂammation [9]. This study showed that SL significantly inhibited p‐p38 and p‐ERK in TNF-α/IFN-γ-induced HaCaT keratinocytes. In summary, *L. rhamnosus* MG4644, *L. paracasei* MG4693, and *Lc. lactis* MG5474 have potential anti-inflammatory effects in TNF-α/IFN-γ-induced HaCaT keratinocytes via the NF‐κB/MAPK signaling pathways. SCFAs, especially propionic and butlyric acid, attenuates TNF-α/IFN-γ-stimulated inflammation in keratinocytes by inhibiting NF-κB activity [39]. Compared to the SCFA production , especially of propionic and butlyric acid of LAB reported in previous studies, *L. rhamnosus* MG4644, *L. paracasei* MG4693, and *Lc. lactis* MG5474 showed high SCFA contents [40]. Thus, these strains showed the most remarkable anti-inflammatory activity in TNF-α/IFN-γ-induced HaCaT keratinocytes, and could be candidate probiotics.

The FAO/WHO Working Group reported that LAB strains should demonstrate probiotic properties, including tolerance under gastrointestinal tract (GIT) conditions and bile salt hydrolase (BSH) de‐conjugation, adhesion to intestinal epithelial cells, and safety (hemolysis and cytotoxicity) [7]. Safety evaluation of LAB strains is the first priority for their use as probiotics because they are rarely used as causative agents of potential opportunistic infections [41]. The MTT assay can measure mitochondrial activity in living cells and is therefore used to measure the cytotoxic effects of samples in vitro [42]. In addition, this assay is widely used as a cytotoxic screening method to evaluate the safety of probiotics [43]. Our results showed that all three strains were non-toxic to intestinal epithelial cells. In addition, hemolysis and BSH activities are considered important safety traits for probiotics [41]. As bile salts are synthesized from cholesterol, some LAB with BSH activity have been suggested to prevent hypercholesterolemia [44]. However, excessive BSH activity can have detrimental effects on the host by causing lipid dyspepsia and impairing colonic mucosal function, potentially leading to the formation of gallstones [45]. In this study, *L. rhamnosus* MG4644, *L. paracasei* MG4693, and *Lc. lactis* MG5474 did not show hemolytic activity nor degrade BSH; therefore, these strains do not harm the host and their safety has been proven.

For consumption as a probiotic, stability in the GIT in the host is important [13]. In other words, probiotics must tolerate bile salt concentrations, enzymes such as pepsin and pancreatin, and low pH (2.5–3.5) [46]. This study found that *L. rhamnosus* MG4644, *L. paracasei* MG4693, and *Lc. lactis* MG5474 survived at more than 95%of the initial viable cell count under simulated GIT conditions. The high survival rate in the simulated GIT is likely to enter the intestine through the gastrointestinal tract when ingested orally by the host [47]. In addition, additional processes, such as microencapsulation, are not required to increase survival rate; so they can be used as economical probiotics. Probiotics, which are short-term colonizers in the epithelial lining, should adhere to the intestinal epidermis and tolerate GIT conditions [45]. As an in vitro model of enterocytes, the bioavailability of HT‐29 cells are of particular interest owing to their similarity to mature enterocytes [48]. In this study, all three strains showed an adhesion rate of > 90% in HT‐29 cells. LAB strains with high adhesion to HT‐29 cells also inhibit pathogens by competitively adhering on the intestinal surface [45]. Thus, *L. rhamnosus* MG4644, *L. paracasei* MG4693, and *Lc. lactis* MG5474 showed tolerance to the GIT by entering the intestine and adhering to the intestinal epithelium, suggesting that they can improve skin inflammation.

## Conclusion

In the current study, *L. rhamnosus* MG4644, *L. paracasei* MG4693, and *Lc. lactis* MG5474 suppressed the production of chemokines and cytokines by inhibiting the NF‐κB/MAPK signaling pathways in TNFα/IFNγ-induced HaCaT keratinocytes. Moreover, these strains have high resistance to GIT conditions, adhered to HT‐29 cells derived from the human colon, and were safe for use by showing no hemolytic or BSH activity. Therefore, *L. rhamnosus* MG4644, *L. paracasei* MG4693, and *Lc. lactis* MG5474 could be used as potential probiotics in functional foods and drugs. However, further in vivo and clinical studies are required.

## Figures and Tables

**Table 1:** The isolates and accession numbers of LAB strains used in this study.

LAB		Accession No.
Human	*L. rhamnosus* MG4288	MW947157.1
	*L. rhamnosus* MG4643	ON668169.1
	*L. rhamnosus* MG4644	ON668170.1
	*L. paracasei* MG4693	OP077096.1
Food	*Lc. lactis* MG5474	ON619520.1
	*Lc. lactis* MG5604	ON705160.1
	*L. reuteri* MG5462	ON619508.1

**Table 2:** SCFA contents in CFS of *L. rhamnosus* MG4644, *L. paracasei* MG4693, and *Lc. lactis* MG5474.

LAB	SCFAs (mg/l)			
	Acetic acid	Propionic acid	Butyric acid	Total
MG4644	2987.00 ± 65.79^ab^	1.49 ± 0.06^a^	5.37 ± 0.04^a^	2993.85 ± 65.69^ab^
MG4693	3112.43 ± 79.35^a^	1.29 ± 0.11^a^	4.83 ± 0.22^a^	3118.55 ± 79.68^a^
MG5474	2643.22 ± 136.40^b^	1.44 ± 0.03^a^	4.97 ± 0.19^a^	2649.63 ± 136.63^b^

Data are presented as mean ± SEM. Different letters (a–d) show significant difference at *p* < 0.05 (*n* = 3).

**Table 3:** Carbohydrate fermentation profiles of *L. rhamnosus* MG4644, *L. paracasei* MG4693, and *Lc. lactis* MG5474.

No.	Type of test^a^	MG4644	MG4693	MG5474	No.	Type of test	MG4644	MG4693	MG5474
1	Control	-	-	-	26	Esculin	+	+	+
2	Glycerol	-	-	-	27	Salicin	+	+	+
3	Erythritol	-	-	-	28	D-Cellobiose	+	+	+
4	D-Arabinose	+	-	-	29	D-Maltose	+	+	+
5	L-Arabinose	-	-	+	30	D-Lactose	+	+	+
6	D-Ribose	+	+	+	31	D-Melibiose	-	-	+
7	D-Xylose	-	-	+	32	D-Sucrose	+	+	+
8	L-Xylose	-	-	-	33	D-Trehalose	+	+	+
9	D-Adonitol	-	-	-	34	Inulin	-	+	-
10	Methyl-β-D-xyloside	-	-	-	35	D-Melezitose	+	+	-
11	D-Galactose	+	+	+	36	D-Raffinose	-	-	+
12	D-Glucose	+	+	+	37	Starch	-	-	+
13	D-Fructose	+	+	+	38	Glycogen	-	-	-
14	D-Mannose	+	+	+	39	Xylitol	-	-	-
15	L-Sorbose	+	-	-	40	Gentiobiose	+	-	+
16	L-Rhamnose	+	-	-	41	D-Turanose	+	+	-
17	Dulcitol	+	-	-	42	D-Lyxose	-	-	-
18	Inositol	+	-	-	43	D-Tagatose	+	+	-
19	D-Mannitol	+	+	-	44	D-Fucose	-	-	-
20	D-Sorbitol	+	-	-	45	L-Fucose	+	-	-
21	Methyl-α-D-mannoside	-	-	-	46	D-Arabitol	-	-	-
22	Methyl-α-D-glucoside	+	+	-	47	L-Arabitol	-	-	-
23	N-Acetylglucosamine	+	+	+	48	Gluconate	+	+	+
24	Amygdalin	+	-	-	49	2-Keto-gluconate	-	-	-
25	Arbutin	+	-	+	50	5-Keto-gluconate	-	-	-

^a^The mark of the result tests: -, not growth; +, growth.

**Table 4:** Enzymatic profile of *L. rhamnosus* MG4644, *L. paracasei* MG4693, and *Lc. lactis* MG5474.

Enzyme assayed for^a^	MG4644	MG4693	MG5474
Control (Negative)	0	0	0
Alkaline phosphatase	1	0	0
Esterase (C4)	3	3	0
Esterase Lipase (C8)	2	3	0
Lipase (C14)	0	0	0
Leucine arylamidase	5	5	5
Valine arylamidase	5	5	2
Crystine arylamidase	2	1	2
Trypsin	0	0	0
α-Chymotrypsin	1	0	1
Acid phosphatase	2	3	3
Naphtol-AS-BI-phosphohydrolase	5	4	2
α-Galactosidase	0	0	0
β-Galactosidase	3	4	0
β-Glucuronidase	0	0	0
α-Glucosidase	2	4	0
β-Glucosidase	5	0	0
N–acetyl -β- glucosaminidase	0	1	0
α-Mannosidase	0	0	0
α-Fucosidase	2	0	0

^a^Data are indicated on scale of 0 (no reaction) to 5 (maximum activity).
